# Carsharing operation optimization with the comprehensive consideration of economic and social benefits

**DOI:** 10.1371/journal.pone.0315323

**Published:** 2025-02-18

**Authors:** Yiping Zhong, Qingyu Luo, Tingting Gao, Yangyang Long, Ling Liu

**Affiliations:** 1 Management College, Jilin Communications Polytechnic, Changchun, China; 2 Transportation College, Jilin University, Changchun, China; 3 FAW Group Co., Ltd, Changchun, China; 4 School of Civil Engineering, Changchun University of Architecture and Civil Engineering, Changchun, Jilin, China; Southwest Jiaotong University, CHINA

## Abstract

Carsharing is regarded as a new mode of transportation that can meet the diversity of travel demands. This study develops a bi-level programming model to design operating strategies aiming to maximize economic and social benefits by determining the site location, number, size and carsharing pricing. In the upper model, a new mixed integer linear programming model is pro-posed to plan the site location, number, size, and pricing of carsharing jointly. Based on the stochastic user equilibrium principle, the lower model is a variational inequality model of travel mode and route choice, to understand the evolution of the transportation system structure after the introduction of shared cars. The results of the case study show the designed scheme can increase the economic benefits of carsharing enterprises and the social benefits of the government, and the site location, number, size, and pricing scheme impact benefits significantly. Furthermore, the setting of shared car sites at public transfer points can promote the interaction between shared cars and other modes of transportation. This study is helpful for carsharing enterprises to increase their economic benefits, enhance their capacity for sustainable growth, and improve the accessibility and flexibility of urban public transportation through sound operational strategies.

## Introduction

In recent years, carsharing has blossomed in urban transportation, changing the travel patterns and structure of urban inhabitants [1]. It reduces the average number of vehicle miles traveled on the road and serves the purpose of easing traffic congestion and saving transportation resources [2]. With the rapid development of shared cars, problems emerge one after another. Firstly, the layout of carsharing sites is inconsistent with the demand, resulting in few available vehicles, low vehicle utilization rate, and difficult parking. Secondly, pricing strategies may induce structural changes in the spatial and journey-purpose profiles of carsharing usage, and affect the user demand and the allocation of resources [3]. Thirdly, carsharing system involves the government, carsharing enterprises, and users. Each subject has different behavior and value pursuit. The policy support of government departments, the standardized operation of carsharing enterprises and the heavy demand of consumers promote the development of carsharing industry.

Therefore, when formulating operational strategies, it is important to consider economic benefits, which can maximize the utilization rate of vehicles and the convenience of users, reduce unnecessary vehicle input and operating costs to ensure that enterprises can balance income and expenditure and achieve profitability, while social benefits are crucial, because it can meet the sustainable development needs of stakeholders such as the government and users. The optimization of carsharing operation can not only bring economic benefits to operators, but also bring value to users and society, and achieve double improvement of economic and social benefits. In addition, the emergence of car sharing has had a significant impact on traditional modes of transportation, and we need to know how the traffic demand structure changes.

The above problems lead to the bottleneck in the development of carsharing. Hence, this study tries to solve them by the following research.

1. To build a bi-level programming model determining the site location, number, size and carsharing pricing with the objective of maximizing economic and social benefits.
2. To provide strategy implications for carsharing enterprises to enhance benefits and expand market size.
3. To propose operation strategy for better interaction between carsharing and other traffic modes.

The rest of the paper is organized as follows. Section 2 reviews the literature related to the study of carsharing operation strategy. Section 3 provides details of the bi-level programming model. Section 4 presents case analysis and scheme comparison. Finally, Section 5 summarizes the study and discusses future research directions.

### Literature review

The carsharing operating problem has been extensively studied across various domains, including operation modes, system optimization, site location, scheduling, and more. The primary objective functions typically focus on maximizing coverage, minimizing total costs, and maximizing benefits. Mixed-integer optimization methods are commonly employed to devise effective strategies for carsharing operations. Researchers have developed models to address different aspects of the problem.

Jorge et al. [4] established a mixed-integer nonlinear programming model to maximize profits and determine the pricing strategy, defined as the journey pricing problem of a single-program vehicle system. Based on the impact of price on vehicle demand, Min Xu et al. [5] built a mixed-integer nonlinear programming model to maximize profit to provide a pricing strategy for shared electric vehicles. Ren et al. [6] designed a dynamic pricing scheme for electric carsharing networks to maximize operator profitability for a data network platform with vehicle network convergence, which changed the demand for carsharing and the travel time between stations, and solved the vehicle imbalance. Mohamed et al. [7] proposed a sharing scheme based on the vehicle dynamic feedback system modeling dynamic pricing method, aiming to control vehicle temporal and spatial imbalance by changing service prices in real-time.

At the same time, they also pointed out the direction to solve the problems of station layout and scheduling. Wang et al. [8] proposed a MILP model to solve the location of carsharing stations, considering vehicle dispatching cost, operator construction cost, and user travel cost, with the lowest total cost as the optimization objective. Chen et al. [9] developed a data-driven MILP model for planning one-way carsharing systems that consider the spatial distribution of demand and the interacting decisions between stations. Correia et al. [10] proposed a mixed-integer optimization method to determine the optimal number of stations, location, and capacity of the one-way carsharing system, whose goal is to maximize the profits of carsharing operators. Correia et al. [11] extended the previous model, which considers trip selection and station location, and felt more alternative pickup points and return points. The results show that user flexibility and vehicle inventory information increase the company’s profits. Zhao and Li [12] proposed a MILP model to determine the optimal allocation of vehicles and personnel to reduce the total cost of carsharing operators. Tan et al. [13] used a competitive game approach to investigate the traveler choice behavior under different pricing strategies and station locations to improve charging stations’ economic efficiency and reliability and obtained the optimal pricing scheme and station distribution. Yoon et al. [14] explored the relationship between potential demand, fleet size, and economic benefits of car sharing in Beijing. The sharing rate of carsharing with different sizes and prices can be determined through the choice preference model. Boyacı [15] used a multi-objective integer linear programming model for a unidirectional carsharing system to optimize the scheduling of vehicles and workers. Antoine et al. [16] proposed a hybrid integer programming model to plan the layout of one-way carsharing stations on a large scale. By dynamically estimating the demand for borrowing and returning cars, the limitation of vehicle distribution imbalance is added to the model. Raposo et al. [17] optimized the location and capacity of electric vehicle charging stations based on a maximum coverage model to maximize demand for a given level of service and budget constraints.

Operation strategy can directly affect users’ willingness to use carsharing, changing travelers’ choice behavior and travel structure. Accurately expressing this relationship can guide the development of a shared car operation strategy. Susan A et al. [18] studied how the story of carsharing will affect the other transportation modes. The results show that the popularity of carsharing will reduce the number of private cars on the road and save maintenance and repair costs for existing or planned remote car users. Jory Firnkorn et al. [19] studied the impact of Car2go, a commercial carsharing model, on reducing private car ownership after it entered the market. They concluded that carsharing had diminished the public’s demand for personal car purchases. Tyndall [20] provided a carsharing layout method based on usage intensity and degree of imbalance. The top results showed that the relationship between the metro and carsharing complemented public transportation. Ceccato et al. [21] used a random forest classifier and a binomial logit model to evaluate the substitution rate of shared cars for private cars and public transport at different prices. The results showed that the pricing scheme could regulate the average substitution rate of shared cars for private cars and public transport. Shi et al. [22] examined how the emergence of ride-hailing services and bike-sharing affected public transportation and how the legalization of ride-hailing services affected their relationship with public transit. The results showed that the emergence of ride-hailing services reduced bus ridership but increased rail ridership. Existing studies on the design of carsharing operation strategies pay much attention on carsharing alone, neglecting the interaction with other transportation modes.

To summarize, there are still the following problems. Firstly, while carsharing generates significant social benefits by improving vehicle utilization, reducing car ownership, easing traffic congestion, and decreasing pollution, these aspects are often overlooked in strategy design from a holistic perspective. Secondly, carsharing operation strategy is inherently an integrated decision problem where changes in one aspect (e.g., pricing) can affect others (e.g., scheduling). Studies that isolate elements like site location, pricing, or scheduling may not yield optimal solutions for the entire system. Finally, many studies focus solely on carsharing without adequately addressing its interaction with other transportation modes.

## Methodology

### Problem description

**Fig 1:**
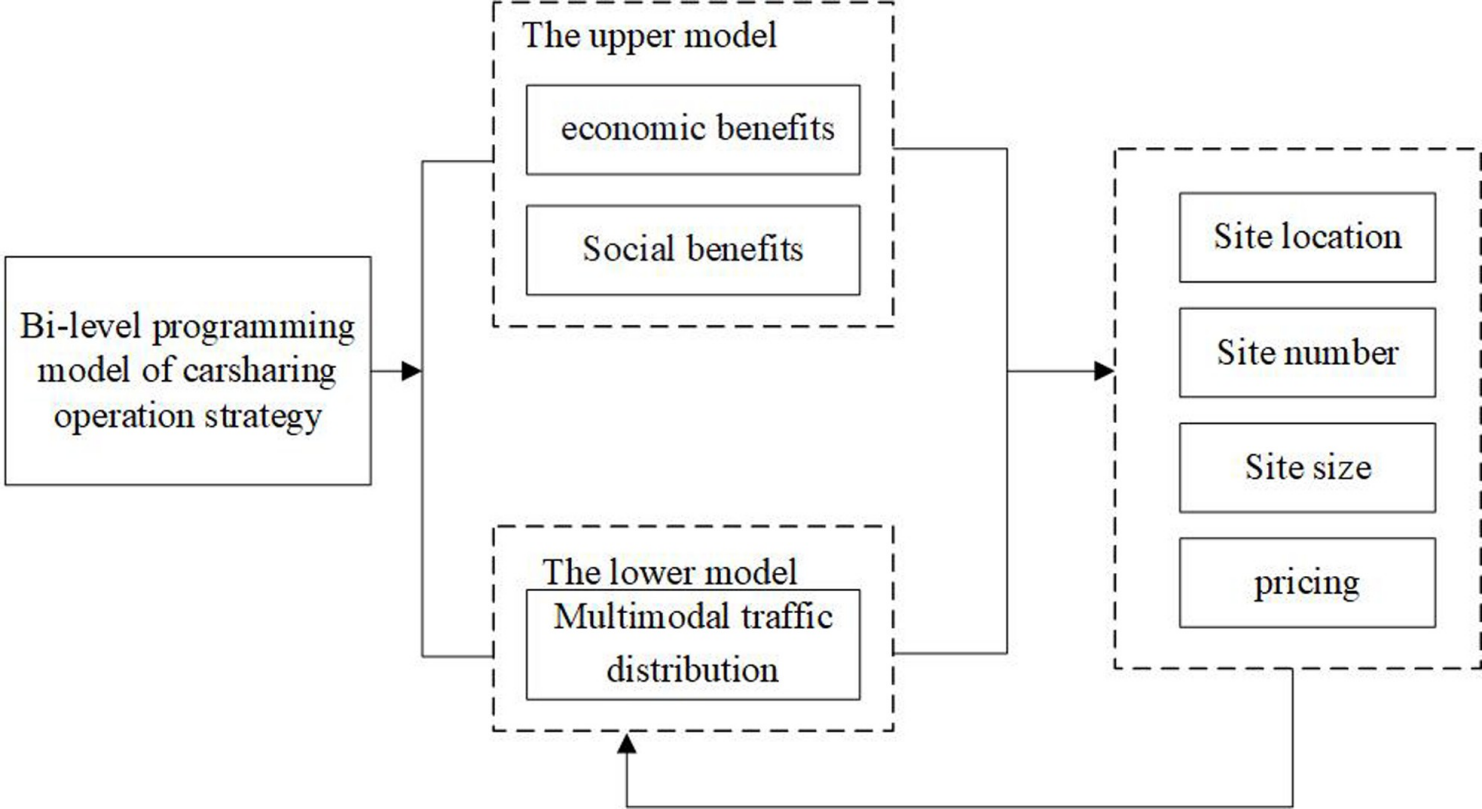
Flow chart of carsharing operation strategy design.

Carsharing is a complex system, and this paper aims at maximizing the sum of the economic benefits of enterprises and the social benefits of the government from a systematic perspective. To solve the problems, such as the inconsistency between layout and requirements, low utilization rate of shared cars, parking difficulties, and the inability to respond to user price sensitivity, a novel carsharing operation strategy is developed, as shown in Fig 1. Due to the large passenger capacity and high social benefits of buses, dedicated bus lanes have been established for buses to study the issue of shared cars. This study focuses on how to plan the initial allocation of a one-way carsharing system, and a bi-level programming model is proposed to solve the site location and pricing of carsharing in multi-mode transportation. The upper model builds a mixed integer linear programming model to plan the layout, number, size, and pricing of carsharing jointly, and the optimization target is maximizing the economic and social benefits. The lower model adopts multi-mode traffic allocation to reflect the impact of shared cars on other traffic modes. In all, after mastering the travel choice behavior in the multi-mode transportation network, the upper decision-makers determine the operation strategy in combination with the expected objectives of stakeholders; in lower decision-making, travelers adjust their travel decisions under operation schemes.

### Model formulation

#### Assumptions

1. All shared vehicles are fuel vehicles.
2. Benefits include economic benefits and social benefits.
3. Carsharing are one-way carsharing, and users can pick up and return the cars at any site.
4. Carsharing and private cars share roads on the urban road network.
5. Buses have independent private lanes, do not interfere with private cars and shared cars.
6. Travelers choose travel modes and routes according to the principle of maximizing random utility, and finally make the multi-mode network reach the equilibrium state.
7. The number of cars initially placed at each site is the same as that of planned parking Spaces.
8. The construction cost of carsharing sites is only related to the number of vehicles on the site.
9. The purchase cost of each vehicle is the same.

#### Notation

*A* = {*A*_*r*_, *A*_*e*_}—Set of real links and virtual links;
*A*_*r*_ = {*I*_*sc*_, *I*_*pc*_}—Set of carsharing and private cars driving on the road;
*A*,*a*—Set of links, link;
*M*,*m*—Set of travel modes, travel mode;
*P*,*p*—Set of travel path, travel path;
*I*—Set of carsharing alternative sites, *I* = {1,2,⋯*i*,⋯};
*u*—Fixed construction costs for a site;
*v*_1_—Construction cost of a single parking space for carsharing, unit: yuan;
*v*_2_—Acquisition cost of shared vehicle, unit: yuan;
*b*—Average number of passengers per shared vehicle;
*φ*_1_—Maintenance costs per mileage traveled by shared vehicles, unit: yuan/km;
*φ*_2_—Dispatch cost per shared vehicle, unit:yuan/veh;
*B*_*i*_—Number of borrowed cars at *i*, unit: veh;
*R*_*i*_—Number of returned cars at *i*, unit: veh;
*C*_*a*_—The travel cost of link *a*;
*x*_*a*_—Traffic flow on link *a*, unit: person/h;
$x_{a}^{sc}$—Carsharing flow on link *a*, unit: person/h;
*L*_*a*_—The length of link *a*,unit: km;
$P_{a}^{sc}$—The cost of driving shared vehicle on real link *a*;
α_1_—Weight of enterprise economic benefits;
α_2_—Weight of government social benefits;
*O*_*I*_—Operating income, unit: yuan;
*OC*_1_—Maintenance cost, unit: yuan;
*OC*_2_—Scheduling cost, unit:yuan;
*C*_*max*_—Maximum number of vehicles in Carsharing site *i*, unit: vehicle;
*C*^*min*^—Minimum number of vehicles in Carsharing site *i*, unit: vehicle
*S*—Social benefit, unit: yuan;
*S*_*c*_—Space benefit, unit: yuan;
*S*_*e*_—Environmental benefit, unit: yuan;
*S*_*p*_—Energy benefit, unit: yuan;
*φ*_*sc*_—The amount of exhaust produced by each shared vehicle, unit: kg(veh∙km);
*ε*_*sc*_—The amount of CO_2_ produced by each shared vehicle, unit:kg(veh∙km);
$\delta_{NO_{x},CO}$—The economic loss caused by poisonous waste gas emitted per unit mass, unit: yuan/kg;
$\delta_{CO_{2}}$—Economic loss caused by CO_2_ emission per unit mass, unit: yuan/kg;
*ξ*_*sc*_—Fuel consumption per kilometer of a carsharing, unit: L/(veh∙km);
*ψ*—The price per liter of fuel, unit: yuan/L;
$\rho_{l}^{SC}$—Decision variable, mileage rates for carsharing, unit: yuan/km;
$\rho_{t}^{Sc}$—Decision variable, time rate for carsharing, unit: yuan/min;
*x*_*i*_—Decision variable, 0–1 variable, if site *i* builds a carsharing site, *x*_*i*_ = 1; otherwise, it is 0;
*r*_*i*_—Decision variable, the number of vehicles placed in the carsharing site *i*;
*q*—Total travel between OD pairs;
*q*^*m*^—The number of trips in mode M;
*h*^*m*^—The expected generalized travel cost of mode M, unit: yuan;
$f_{p}^{m}$—The travel volume of mode M on path P, unit: person⁄h;
$C_{p}^{m}$—The generalized travel cost of mode M on path P, unit: yuan;
$x_{a}^{m}$—Traffic flow of mode M on link *a*, unit: person⁄h;
*λ*_1_—The sensitivity of travelers’ mode choice to travel cost, known constant;
*λ*_2_—The sensitivity of travelers’ route choice to travel cost, known constant;
*δ*_*a*,*p*_—Path-link indicator variables for path *p*,if the path *p* after link *a*,*δ*_*a*,*p*_ = 1, otherwise *δ*_*a*,*p*_ = 0.

#### The upper model

1. Economic benefits of operators

Carsharing enterprises hope to achieve the maximum economic benefits with operating strategies. The economic benefits are related to the cost invested in site construction, the purchase of vehicles, and the operating income and costs in the operation. The economic benefit is the difference between operating revenue and operating cost. Operating income (OI) is the fee paid by users of shared cars, using a dual-rate billing system according to travel time and mileage. The operating income is obtained by multiplying the carsharing traffic of each link with the monetary cost.


$$OI=\sum_{a}P_{a}^{sc}\left(\frac{x_{a}^{sc}}{b}\right),a\in A_{r},A_{r}\in I_{sc}$$ (1)

The link cost is calculated from the actual link cost of shared vehicle in the generalized road travel costs, as follows

$$P_{a}^{sc}=\rho_{l}^{sc}L_{a}+\rho_{t}^{sc}T_{a},a\in A_{r},A_{r}\in I_{sc}$$ (2)

The carsharing enterprises’ operation cost includes the initial investment cost, maintenance cost, and scheduling cost during operation.

The distribution and scale of carsharing sites are limited by the cost of land, labor, and materials needed in construction. Moreover, the purchase of vehicles is also expensive. Therefore, the initial investment costs contain site construction costs and vehicle purchase costs. To enter the market, carsharing enterprises inevitably need to invest in the initial cost. The investment is significant, and the payback period is long. Therefore, economic benefits only focus on operating profit and take the initial investment cost as the constraint condition of capital investment.

The shared cars in this study are fuel cars, and enterprises need to replenish fuel according to vehicle usage during operation. In addition, vehicle wear occurs when the vehicle is running, and the enterprise is responsible for the maintenance and cleaning of the car, resulting in maintenance costs. Therefore, the maintenance cost caused by the wear and tear of fuel-consuming vehicles needs to be considered. Maintenance costs (*OC*_*1*_) is positively correlated with mileage, expressed as a function of mileage.


$$OC_{1}=\varphi_{1}L_{a}\sum_{a}\left(\frac{x_{a}^{sc}}{b}\right)$$ (3)

One-way carsharing allows users to borrow and return cars between different sites. Due to vehicle movement between locations, there is a mismatch between the number of cars at a site and user demand. Therefore, carsharing enterprises should make the demand for borrowing and returning at sites as balanced as possible to reduce scheduling costs. The scheduling cost (*OC*_*2*_) is related to the difference of the number of vehicles borrowed and returned at sites, and is expressed as a function of the difference of vehicles borrowed and returned.


$$OC_{2}=\varphi_{2}\sum_{i}\left|B_{i}-R_{i}\right|,i\in n_{i}$$ (4)

Where the number of borrowed vehicles at site *i* is the sum of the carsharing traffic on the online link and the number of returned vehicles is the sum of the carsharing traffic on the off-grid link.


$$B_{i}=\frac{\sum_{a\in \left(,n_{i}\right)}x_{a}^{sc}}{b},\left(x_{a}^{sc},a\in A_{e1}\right)$$ (5)

$$R_{i}=\frac{\sum_{a\in \left(n_{i}\right)}x_{a}^{sc}}{b},\left(x_{a}^{sc},a\in A_{e3}\right)$$ (6)

Operating cost (*OC*) is the sum of maintenance cost and scheduling cost.


$$OC=\varphi_{1}L_{a}\sum_{a}\left(\frac{x_{a}^{sc}}{b}\right)+\varphi_{2}\sum_{i}\left|B_{i}-R_{i}\right|,a\in A_{r},A_{r}\in I_{sc},i\in n_{i}$$ (7)

The operator’s economic benefit can be formulated as follows:

Y=(OI-OC) (8)

2. Social benefit of the government

Carsharing is expected to reduce private car ownership, promote car utilization, guide travelers to use public transportation, alleviate urban traffic congestion, reduce exhaust emissions, and save social energy. Therefore, social benefits are essential to fulfill the needs of stakeholders, such as the government and users, for sustainable development. Social benefits can be expressed as the sum of spatial benefit *S*_*c*_, environmental benefit *S*_*e*_ and energy benefit *S*_*p*_.


$$S=S_{c}+S_{e}+S_{p}$$ (9)

Spatial benefits refer to the occupation saving of urban space road when some users choose shared cars instead of private cars.


$$S_{c}=\sum_{a}\left[C_{a}^{sc}\left(\frac{x_{a}^{sc}}{b}\right)-C_{a}^{pc}x_{a}^{pc}\right],a\in A_{r},A_{r}\in \left[I_{pc},I_{sc}\right]$$ (10)

Environmental benefits refer to the reduction in pollution caused to the urban environment when some users choose car sharing instead of private car.


$$S_{e}=\sum_{a}\delta_{NO_{x},CO}\times \varphi_{c}L_{a}\left[\left(\frac{x_{a}^{sc}}{b}\right)-x_{a}^{pc}\right]+\delta_{CO_{2}}\times \varepsilon_{c}L_{a}\left[\left(\frac{x_{a}^{sc}}{b}\right)-x_{a}^{pc}\right]$$ (11)

Where φ_*c*_ is defined as emissions per kilometer produced by a single carsharing, *ε*_*c*_ as the CO_2_ per kilometer for carsharing, $\delta_{NO_{x},CO}$ and $\delta_{CO_{2}}$ are respectively for the economic loss caused by unit quality toxic exhaust and CO_2_ emission.

Since the per capita energy consumption of private cars is higher than that of shared cars, the energy benefit is defined as the reduction in energy consumption when some users choose to travel in shared cars.


$$S_{p}=\sum_{a}\frac{\psi }{100}\xi_{c}L_{a}\left[\left(\frac{x_{a}^{sc}}{b}\right)-x_{a}^{pc}\right]$$ (12)

Social benefits can be expressed as

$$S=\sum_{a}\left[\begin{array}{l} \left[C_{a}^{sc}\left(\frac{x_{a}^{sc}}{b}\right)-C_{a}^{pc}x_{a}^{pc}\right]+\delta_{NO_{x},CO}\times \varphi_{c}L_{a}\left[\left(\frac{x_{a}^{sc}}{b}\right)-x_{a}^{pc}\right.\\ +\delta_{CO_{2}}\times \varepsilon_{c}L_{a}\left[\left(\frac{x_{a}^{sc}}{b}\right)-x_{a}^{pc}\right]+\frac{\psi }{100}\xi_{c}L_{a}\left[\left(\frac{x_{a}^{sc}}{b}\right)-x_{a}^{pc}\right] \end{array}\right]$$ (13)

Based on the above analysis, the upper target is the maximum sum of economic benefits and social benefits with different weights. The upper model is defined as

$$C=max\left(\alpha_{1}Y+\alpha_{2}S\right)$$ (14)

S.t

$$\sum_{i\in n_{i}}ux_{i}+\sum_{i\in n_{i}}\left(v_{1}+v_{2}\right)r_{i}x_{i}\leq W$$ (15)

$$C_{min}x_{i}\leq r_{i}\leq C_{max}x_{i},\forall i\in I$$ (16)

1≤∑i≤I (17)

$$r_{i}>0,if\left(x_{i}=1\right)$$ (18)

$$B_{i}<Mx_{i},\forall i\in I$$ (19)

$$R_{i}<Mx_{i},\forall i\in I$$ (20)

$$x_{i}\in \{0,1\},\forall i\in I$$ (21)

$$r_{i}\in Z^{+},\forall i\in I$$ (22)

$$\rho_{l}^{sc},\rho_{t}^{sc}\geq 0$$ (23)

Constraint (15) indicates that the initial investment of carsharing site construction should not exceed the maximum acceptable cost for operators. Most carsharing sites are rebuilt from existing parking lots, roadside parking spots, and vacant lots. One part of the construction cost is fixed costs such as rent. Another part is the variable cost required to transform each parking spot, which is positively correlated with the size of the parking space. The larger the scale, the more the construction cost. The vehicle purchase cost is proportional to the number of vehicles, and the number of vehicles is supposed to be the same as the number of planned parking spots. The vehicle purchase cost and construction variable cost are expressed together here.

Constraint (16) indicates that the number of vehicles at site *i* cannot exceed the limit of site size. Constraint (17) indicates that the number of selected sites is greater than or equal to 1 and less than or equal to the number of alternative sites. Constraint (18) indicates that if site i is selected, the site size must be greater than zero. Constraint (19)-(20) indicate that carsharing users can only borrow and return cars at the planned sites. Constraints (21)-(23) represent the value range of decision variables.

#### The lower model

1. The impact of carsharing on traffic distribution

- Number of vehicles deployed at sites

**Fig 2:**
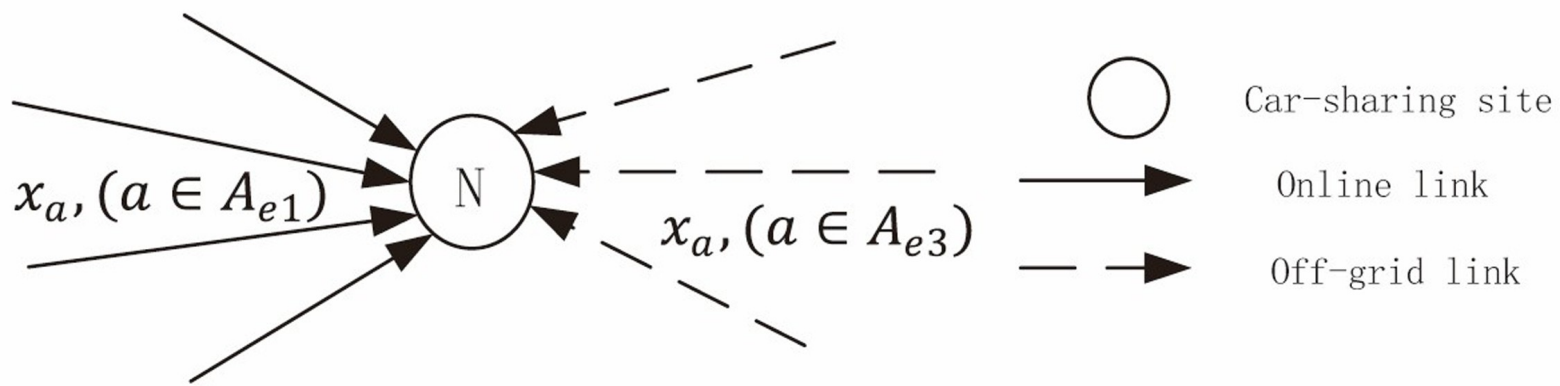
Relationship of car borrowing and returning demand and link flow of carsharing sites.

The demand for car sharing is increasing, but the number of vehicles available at sites is limited. When users arrive at the sites, their demand for a car may not be met immediately, resulting in queues. Limitations on the number of vehicles placed at sites must be considered in the traffic allocation. Fig 2 shows the relationship between car borrowing and returning demand and link traffic flow of carsharing site. A queuing model can be established to describe the queuing situation of users at sites.

The input of the queuing model is related to the link flow. For a particular carsharing station, the car borrowing demand is the sum of the traffic of all online links connected to the site (The online links indicate that the traveler arrives at the multi-modal network from the origin of the trip). The car returning demand is the sum of the traffic on all the off-grid links connected to the station (The off-grid links indicate that the traveler leaves the multi-modal network to reach the destination).

Assuming that *x*_*a*_ is the traffic volume of the link *a*, *b* is the average number of passengers per vehicle, *B* is the demand for borrowing vehicles at the site, *R* is the demand for returning vehicles at the site, and *r* is the number of vehicles released at the site, then

$$B=\frac{\sum_{a}x(a)}{b},a\in A_{e1}$$ (24)

$$R=\frac{\sum_{a}x(a)}{b},a\in A_{e3}$$ (25)

**Fig 3:**
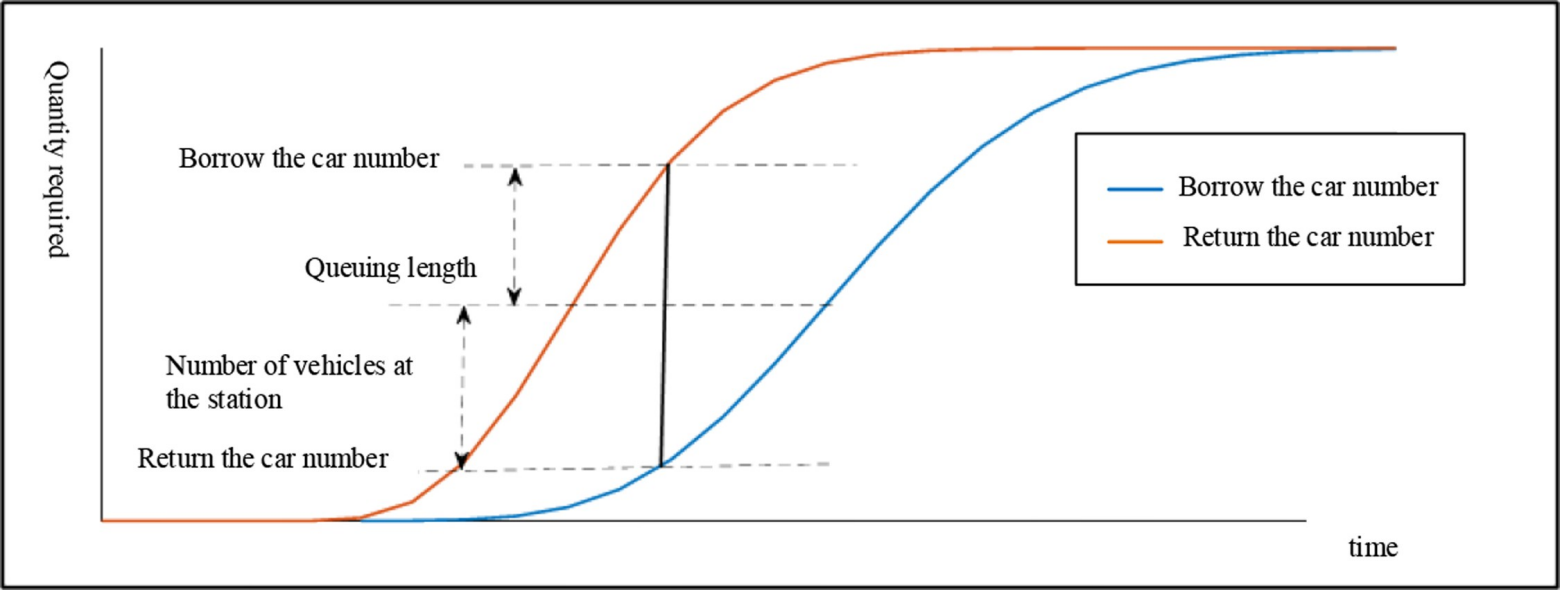
Users borrowing and returning car demand curve.

Users adhere to the first-come, first-served queuing rule. The rule is a wait-loss system. When users arrive at the station, they will wait in a queue if there is no available vehicle, and the waiting time will be lost. The carsharing site has multiple vehicles, and each vehicle can be taken as a service station. The queuing model is a multi-service station queuing system. The number of service desks is the number of vehicles, and the distribution of service hours is the distribution of vehicle usage time. In the queuing process, the number change curve of cars borrowed and returned by users is shown in Fig 3.

According to the queuing model, the average queuing length can be expressed as

$$L=\sum_{B=r+1}^{\infty }\left(B-r\right)P_{B}$$ (26)

$$P_{B}=\left\{\begin{array}{c} \frac{1}{B!}{\left(\frac{\lambda }{\mu }\right)}^{B}P_{0},(B\leq r)\\ \frac{1}{r!r^{B-r}}{\left(\frac{\lambda }{\mu }\right)}^{B}P_{0},(B>r) \end{array}\right.$$ (27)

$$P_{0}=\sum_{k=0}^{r-1}{\left[\frac{1}{k!}{\left(\frac{\lambda }{\mu }\right)}^{k}+\frac{1}{r!}\frac{r\mu }{r\mu -\lambda }{\left(\frac{\lambda }{\mu }\right)}^{r}\right]}^{-10}$$ (28)

*P*_B_ is the expected wait time of arrival, *P*_0_ is the probability of 0 users in the carsharing system, *λ* is the average arrival speed of the user, *μ* is the average vehicle speed of users.

The average waiting time of users arriving at the carsharing site is

$$t=\frac{L}{\lambda }$$ (29)

- Distance from sites to origins and destinations

One-way carsharing sites have limited coverage, and not all origins and destinations are available for carsharing trips. As a result, the distance from the trip’s origin and destination to the carsharing site must be considered. It is assumed that the maximum acceptable walking distance of carsharing users looking for vehicles is *s*. A circle is drawn with the starting and ending points as the center and s is taken as the radius to judge whether an OD pair can use carsharing. If there are carsharing sites within the acceptable walking distance from the starting and ending points, they can choose the sites to travel; otherwise, travelers cannot use them.

**Fig 4:**
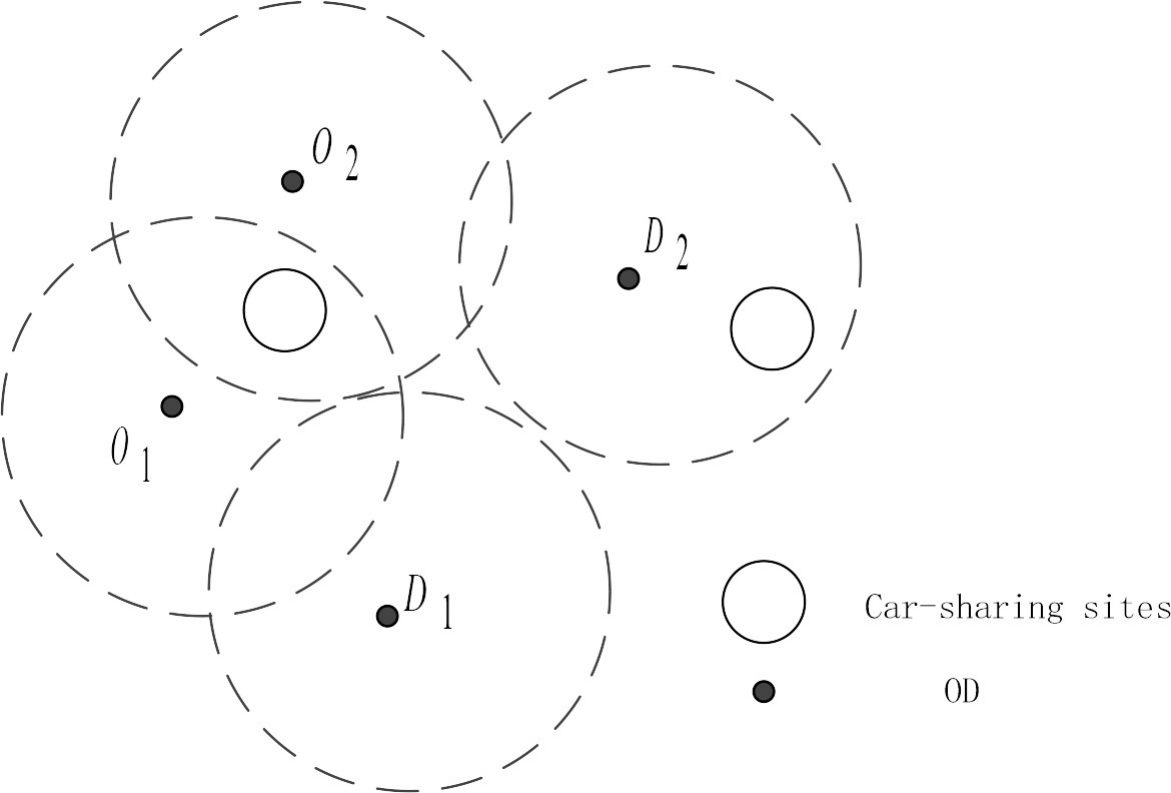
Distance diagram from the starting and ending points to carsharing sites.

As shown in Fig 4, for the point {O_1_, D_1_}, there are carsharing sites within the acceptable walking distance of the starting point O_1_, then they can be used for travel. However, there are no carsharing sites within the acceptable walking distance of destination D_1_, so carsharing cannot be selected. For the starting and ending points {O_2_, D_2_}, there are carsharing sites within the acceptable walking distance of the starting and ending points, so travelers can choose carsharing to travel.

The effective path search algorithm can solve the influence of the distance from sites to origins and destinations on traffic assignment. If the trip origin and destination do not satisfy the distance constraint, the path is removed from the valid paths. The specific steps to check the distance constraint of carsharing sites are as follows. 1) Determine whether the initial link of the path is the online access link of carsharing. If so, delete the path that does not meet the distance constraint; otherwise, retain the path. 2) Determine whether the terminated link of the path is the link of carsharing off the network. If so, delete the path that does not meet the distance constraint; otherwise, retain the path.

2. Lower model building

In multi-mode traffic network, mode selection and traffic distribution are carried out according to Wardrop first equilibrium principle (user equilibrium principle). In the trip, the traveler has to make both the travel mode choice and the travel path choice. When a multi-modal network approaches equilibrium, all travelers are unable to unilaterally modify their travel mode or travel path to minimize their desired generalized travel cost. At this point, all modes and paths have the exact generalized travel cost. Travelers between OD pairs make mode and route selection with a random error term of independent homologous Gumbel distribution. Drawing on the random effects theory and the Logit model, the choice behavior of travelers is given. The multi-modal network mode division and traffic assignment equilibrium model satisfying the stochastic user balance principle can be expressed as

$$q^{m}=q\frac{exp\left[-\lambda_{1}h^{m}\right]}{\sum_{m}exp\left[-\lambda_{1}h^{m}\right]}$$ (30)

$$h^{m}=-\left(\frac{1}{\lambda_{2}}\right)ln\left(\sum_{p\in P^{m}}exp\left(-\lambda_{2}C_{p}^{m}\right)\right)$$ (31)

$$f_{p}^{m}=q_{rs}^{m}\frac{exp\left[-\lambda_{2}C_{p}^{m}\right]}{\sum_{p\epsilon P^{m}}\left[-\lambda_{2}C_{p}^{m}\right]}$$ (32)

$$\sum_{p\in P}f_{p}^{m}=q^{m},m\in M$$ (33)

$$\sum_{m\in M}q^{m}=q_{rs}$$ (34)

$$f_{p}^{m}\geq 0,p\in P$$ (35)

$$q^{m}\geq 0$$ (36)

$$x_{a}^{m}=\sum_{p\in P}f_{p}^{m}\delta_{a,p},a\in A,m\in M$$ (37)

$$x_{a}=\sum_{m\in M}x_{a}^{m},a\in A$$ (38)

Considering the stochastic user equilibrium principle, the lower model is constructed to be a variational inequality model of travel mode and route choice.


$$\sum_{m\in M}\sum_{p\in P^{m}}\left[C_{p}^{m}\right.\left.+\frac{1}{\lambda_{2}}\ln\left(\frac{f_{p}^{m*}}{q^{m*}}\right)\right]\left(f_{p}^{m}-f_{p}^{m*}\right)+\sum_{m\in M}\frac{1}{\lambda_{1}}\ln\frac{q^{m*}}{q}\left(q^{m}-q^{m*}\right)\geq 0$$ (39)

The feasible region of the model satisfies the constraint conditions (33)-(38), $f_{p}^{m*},q^{m*}$ are the optimal solution obtained by traffic assignment.

Theorem 1 The variational inequality model (39) is equivalent to the stochastic user equilibrium principle expressions (30)-(32) in multiple modes.

Proof: From the KKT condition of the variational inequality model (39), we can obtain

$$C_{p}^{m}+\frac{1}{\lambda_{2}}ln\left(\frac{f_{p}^{m}}{q^{m}}\right)-l^{m}-\phi_{p}^{m}=0$$ (40)

$$\frac{1}{\lambda_{1}}ln\frac{q^{m}}{q}+l^{m}-l=0$$ (41)

$$f_{p}^{m}\phi_{p}^{m}=0$$ (42)

$$\phi_{p}^{m}\geq 0$$ (43)

Where $\phi_{p}^{m},l^{m}$ and *l* are $f_{p}^{m},q^{m}$ and qdual variable, respectively.

From Eq (40), $f_{p}^{m}$>0. Substitute Eqs (42) and (43) into Eq (40), get

$$\frac{f_{p}^{m}}{q^{m}}=exp\left[\lambda_{2}\left(l^{m}-C_{p}^{m}\right)\right]=exp\left(\lambda_{2}l^{m}\right)\times exp\left(\lambda_{2}C_{p}^{m}\right)$$ (44)

Sum the path *P*, combine with constraint (33), get

$$exp\left(\lambda_{2}l^{m}\right)\sum_{p\in P}exp\left(-\lambda_{2}C_{p}^{m}\right)=1$$ (45)

It can be introduced that *l*^*m*^ is the minimum expected travel cost for the traveler’s choice of mode *M* in Eq (41)). Substituting Eqs (45) into (44), we obtain the path choice logit model Eq (32).

Similarly, the logit model of mode choice (30) can be obtained from Eqs (41) and (34).

Theorem 2 The variational inequality model (39) has at least one solution.

Proof: The constraints (33)-(38) of the variational inequality model (39) are a set of linear constraints. Functions $C_{p}^{m},ln\left(\frac{f_{p}^{m}}{q^{m}}\right),ln\frac{q^{m}}{q}$ are continuous functions, according to Brouwer fixed point theorem, it can be inferred that the model has at least one solution.

### Solution algorithm

#### NSGA-II algorithm for feasible schemes

The carsharing strategy is a mixed-integer nonlinear programming problem, which belongs to the NP-hard problem and is solved by a heuristic algorithm. The non-dominated sorting genetic algorithm with elite strategy (NSGA-II) is selected to solve the bi-level optimization problem. This algorithm has the advantages of solid global searchability, good compatibility, and high fault tolerance. The core is to coordinate the relationship of each objective function and find the optimal solution set that makes the objective reach the maximum possible value. The algorithm has two essential mechanisms: non-dominated sorting and congestion calculation.

Since there are both 0–1 variables and real variables in the decision variables, the mixed coding method of binary encoding and real encoding is adopted. Binary encoding is adopted for 0–1 variables and real encoding is adopted for real variables. The algorithm steps are as follows.

Step 0: Set the population size as N, randomly generate the initial population P_t_, and let the number of iterations t = 0.
Step 1: Calculate the lower-level model. Substitute the population into the lower-level model, use MSA algorithm to solve ${\text{x}}_{\text{a}}^{\text{m}}$, then substitute into the upper level to find the value of the objective function.
Step 2: Non-dominated sort. According to the objective function value, the non-dominated sort is performed on P_t_, and the rank value of each individual is recorded.
Step 3: Select, cross, and mutate. A selective cross mutation operation is performed on P_0_ to generate the first generation of offspring population Q_t_, and let the iteration times t = 1.
Step 4: Merging parent and child. Populations merging P_t_ and Q_t_ to produce combined population R_t_ = P_t_ ∪ Q_t_.
Step 5: Generation of new parent population. According to the objective function value, the rapid non-dominated sort crowding degree of R_t_ is calculated, and N individuals are selected through the elite retention strategy to form a new parent population P_t_.
Step 6: Select, cross, and mutate. A selective cross mutation operation is performed on P_t_ to generate the first generation of offspring population Q_t_.
Step 7: Stop. Judging t ≤ max (t). If so, make *t* = *t* + 1, jump to step Step1; otherwise, end the loop.

#### Scheme comparison based on entropy weight TOPSIS method

A certain number of site schemes can be output from the lowest to the highest by solving the model. The site investment intensity and traffic accessibility of the shared car sites can be calculated separately for each option based on the site selection. The scheme comparison will be evaluated by benefit value, site investment intensity, and traffic accessibility of carsharing stations.

Investment intensity is generally defined as fixed asset investment per unit area within the construction project site. Site investment intensity can reflect the regional economic development level, investment, and operation environment. It has a significant impact on the selection of carsharing sites. Site investment intensity can be expressed as

$$C_{Z}=F/S$$ (46)

Where *C*_*Z*_ is the investment intensity of alternative sites in cities where carsharing are located, unit: yuan/m^2^,*F* is the total amount of fixed assets invested in the shared site in the city, unit: yuan, *S* is the construction land area of the city where stations are located, unit: m^2^.

When users choose to use a shared car, the site needs to meet the user’s needs for borrowing and returning. Good accessibility is a guarantee to increase the use of car sharing. The layout of carsharing stations should meet a wide range of user demands and be deployed at public transportation interchange points to facilitate the connection with the public. Highly accessible nodes with more convenient transportation are more suitable as a carsharing site.

The accessibility between stations in a multi-modal transportation network is determined by a matrix-based topology approach. Specifically, it is assumed that *T* is the adjacency matrix that show the connectivity of carsharing between sites. (The value of the element in the matrix is 1, which means connected, and 0, which means disconnected). It can be proved by the rules of matrix operation, $t_{ij}^{\left(k\right)}$ in the kth power *T*^*k*^of the matrix *T* is the number of nodes *j* that can be reached from node *i* using shared car after *k*(*k* = 1,2,……, *n*)steps. The *D* =*T*^1^ + *T*^2^ +⋯+*T*^n^, If the number of nodes in the network is *N*, the total number of nodes that node *i* can reach directly and indirectly is $d_{i}=\sum_{j=1}^{N}d_{ij}$. Finally, it is concluded that node *i* adopts the relative accessibility index *D*_*i*_ of shared vehicles.


$$D_{i}=\frac{d_{i}}{\sum_{i=1}^{N}\frac{d_{i}}{N}}$$ (47)

After calculating the investment intensity and the accessibility of carsharing sites respectively, the original data matrix Table 1 can be summarized by combining the output results of the optimization model.

**Table 1:** Raw data matrix.

Scheme number	Efficiency value	Site investment intensity	Accessibility of sites
1	x_11_	*x*_12_	*x*_13_
2	x_21_	*x*_22_	*x*_23_
3	x_31_	*x*_32_	*x*_33_
4	x_41_	*x*_42_	*x*_43_
…	…	…	…
n	x_n1_	*x*_*n*2_	*x*_*n*3_

The weight of each index is determined by entropy method, and the steps are as follows.

Step 1: Standardize raw data matrix. That is, the negative index in the original matrix *X* is positive and dimensionless. The *j*th index value of the standardized scheme *p*_*ji*_ ∈ [0,1], the indicator matrix is the matrix *P*.
Step 2: Calculate the entropy of each index *H*(*x*_*j*_):
$$H\left(x_{j}\right)=-k\sum_{i=1}^{n}p_{ij}\ln p_{ij}$$ (48)
  Where, *k* is the adjustment coefficient, $k=\frac{1}{ln(n)}>0$.
Step 3: Calculate the index difference coefficient *h*_*j*_:
$$h_{j}=1-H\left(x_{j}\right)$$ (49)
Step 4: Determine the weight coefficient of each index *W*_*j*_:
$$W_{j}=\frac{h_{j}}{\Sigma_{j=1}^{n}h_{j}}$$ (50)

The best scheme selection is based on TOPSIS method as follows.

Step 1: The normalized matrix is multiplied by the index weight matrix to obtain the index matrix considering the weight Z:
$$Z=P\cdot W=\left[\begin{array}{ccc} z_{11} & z_{12} & z_{13}\\ z_{21} & z_{22} & z_{23}\\ \cdots & \cdots & \cdots \\ z_{n1} & z_{n2} & z_{n3} \end{array}\right]$$ (51)
Step 2: Find the maximum and minimum values of each index in matrix Z, and construct the optimal solution vector *Z*_*max*_ and the worst solution vector *Z*_*min*_.
$$Z_{max}=\left[\begin{array}{llll} z_{r1} & z_{r2} & \cdots & z_{rn} \end{array}\right]$$ (52)
$$Z_{min}=\left[\begin{array}{llll} z_{w1} & z_{w2} & \cdots & z_{wn} \end{array}\right]$$ (53)
  Among them, *z*_*rj*_ and *z*_*wj*_ are the optimal value and worst value of the jth column index respectively.
Step 3: Calculate the distance between each scheme and the optimal solution (the worst solution), respectively
$$D_{i}^{+}=\sqrt{\sum_{j}^{n}{\left(z_{max\;j}-z_{ij}\right)}^{2}}$$ (54)
$$D_{i}^{-}=\sqrt{\sum_{j}^{n}{\left(z_{min\;j}-z_{ij}\right)}^{2}}$$ (55)
  Finally, the calculation formula of relative closeness, which represents the merits and demerits of the scheme, is
$$C_{i}=\frac{D_{i}^{-}}{D_{i}^{+}+D_{i}^{-}}$$ (56)

The solution with the highest closeness is the optimal solution under the multi-objective decision of integrated benefit value, station investment intensity and accessibility of carsharing stations.

### Case study

**Fig 5:**
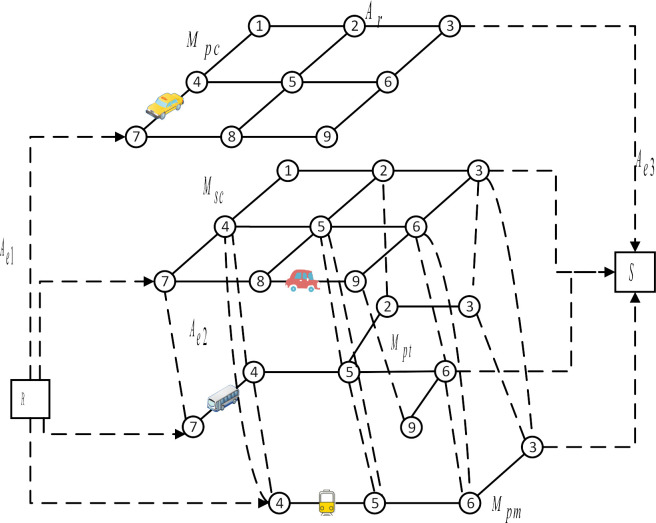
A multimodal transportation network with carsharing.

The multi-mode traffic network constructed in Fig 5. is used for example analysis to design the operation strategy. It consists of 1 OD point pair and 4 transportation mode sub-networks (private, carsharing, bus and subway), which are connected by transfer links.

**Fig 6:**
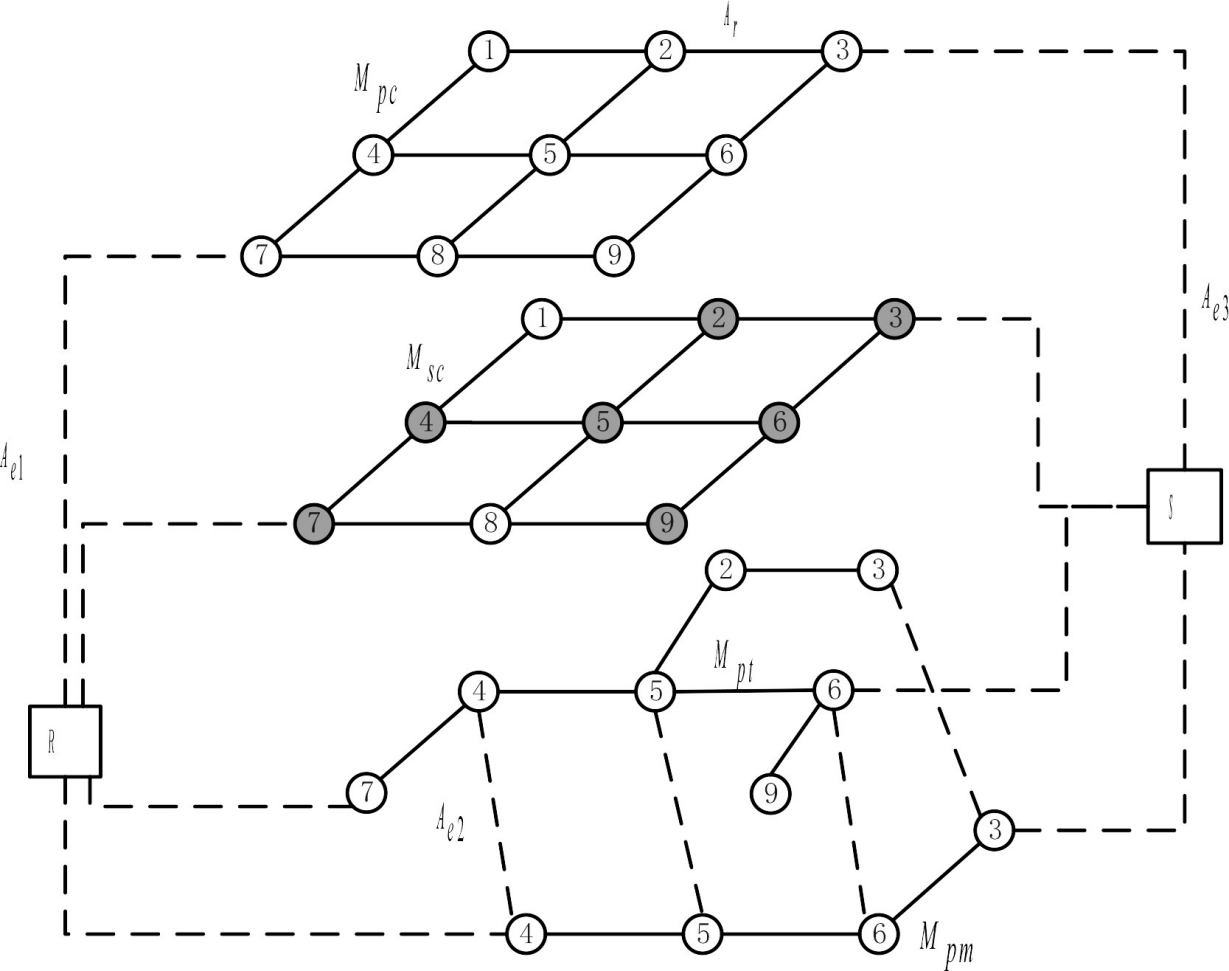
Test road network.

According to the principle of site selection, nodes 7 and 3, near to travel demand locations, and nodes 2, 3, 4, 5, 6, and 9, which can be interchanged with public transit, are chosen as an alternate site set in the carsharing network. The network structure is shown in Fig 6. Tables 2 and 3 show the specific road network information. The road network contains 1 OD pair, and the OD traffic volume is 3000 person/h.

**Table 2:** Road network information.

Road link	Link length (km)	The car has zero flow time (min)
(1,2)	4	5
(2,3)	6	8
(4,5)	5	5
(5,6)	6	8
(7,8)	6	7
(8,9)	5	5
(1,4)	8	11
(2,5)	6	8
(3,6)	8	12
(4,7)	5	6
(5,8)	6	8
(6,9)	6	7

**Table 3:** Virtual link information.

Link owning set	Stretch properties	Road link		Link length (m)
Set of online links	Private car	(*R*,(7,*pc*))		0
	Carsharing	(*R*,(7,*sc*))		200
	Bus	(*R*,(7,*pt*))		300
	Subway	(*R*,(4,*pm*))		1200
Set of interchange links	Carsharing transfers with bus	{(2,*sc*),(2,*pt*)}	{(2,*pt*),(2,*sc*)}	80
		{(3,*sc*),(3,*pt*)}	{(3,*pt*),(3,*sc*)}	50
		{(4,*sc*),(4,*pt*)}	{(4,*pt*),(4,*sc*)}	45
		{(5,*sc*),(5,*pt*)}	{(5,*pt*),(5,*sc*)}	55
		{(6,*sc*),(6,*pt*)}	{(6,*pt*),(6,*sc*)}	50
		{(7,*sc*),(7,*pt*)}	{(7,*pt*),(7,*sc*)}	70
		{(9,*sc*),(9,*pt*)}	{(9,*pt*),(9,*sc*)}	60
	Carsharing transfers with subway	{(3,*sc*),(3,*pm*)}	{(3,*pm*),(3,*sc*)}	60
		{(4,*sc*),(4,*pm*)}	{(4,*pm*),(4,*sc*)}	60
		{(5,*sc*),(5,*pm*)}	{(5,*pm*),(5,*sc*)}	45
		{(6,*sc*),(6,*pm*)}	{(6,*pm*),(6,*sc*)}	50
	Bus transfers with subway	{(3,*pt*),(3,*pm*)}	{(3,*pm*),(3,*pm*)}	200
		{(4,*pt*),(4,*pm*)}	{(4,*pm*),(4,*pm*)}	150
		{(5,*pt*),(5,*pm*)}	{(5,*pm*),(5,*pm*)}	180
		{(6,*pt*),(6,*pm*)}	{(6,*pm*),(6,*pm*)}	200
Set of off-grid links	Private	((3,*pc*),*S*)		0
	Carsharing	((3,*sc*),*S*)		200
	Bus	((3,*pt*),*S*)		500
	Subway	((3,*pm*),*S*)		500

To validate the bi-level planning for operation strategy, combine practice and literatures [23,24], obtain data through actual investigation, the parameter values of the upper model are shown in Table 4, and the parameter values of the lower model are shown in Table 5. The decision variables in the upper model are scoped based on an examination of current carsharing enterprises and pricing schemes. The number of vehicles placed at a single site is defined from 10 to 20, and the mileage rate $\rho_{1}^{sc}$ ranges from 0.1 to 1.0yuan/km and time rate $\rho_{\text{t}}^{\text{sc}}$ ranges from 0.1 to 1 *yuan/min*.

**Table 4:** Parameter value table of the upper model.

Parameter	Implication	Unit	Value
*φ*_1_	Maintenance cost per mile traveled	yuan/km	0.1
*φ*_2_	The scheduling cost	yuan/veh	10
*u*	Site fixed construction costs	yuan	3×10^4^
*v*_1_	Construction cost of a single parking space	yuan	2×10^3^
*v*_2_	Vehicle acquisition cost	yuan	5×10^4^
*W*	Maximum acceptable cost for the operator	yuan	5×10^6^
*γ* ^*c*^	Coefficient of time value	-	1.0
*φ*_*sc*_	Emissions per kilometer produced by a single carsharing	*kg*/(*veh∙km*)	0.213
*ε*_*sc*_	The amount of *CO*_2_ per kilometer produced by a single carsharing	*kg*/(*veh∙km*)	0.248
$\delta_{NO_{x},CO}$	Economic loss caused by waste gas emitted per unit of mass	*yuan*/*kg*	78.54
$\delta_{CO_{2}}$	Economic loss caused by emission unit mass *CO*_2_	*yuan/kg*	0.23
*ξ*_*sc*_	Fuel consumption of carsharing	*L*/(*veh∙km*)	10
*ψ*	The price per liter of fuel	*yuan*/*L*	8.0
*φ*_1_	Maintenance cost per mile traveled	yuan/km	0.1
*φ*_2_	The scheduling cost	yuan/veh	10

**Table 5:** Parameter value table of the underlying model.

Parameter	Implication	Value	Parameter	Implication	Value
*α*	-	0.15	*β*	-	4
*ω*_1_	Currency-time conversion coefficient	2	*ω*_2_	Comfort-time conversion coefficient	0.5
*ρ*_*w*_	Comfort loss per unit waiting time	0.2	*ρ*_*f*_	Comfort loss per unit walking time	1
*λ*_1_	Mode selection sensitivity coefficient	0.1	*λ*_2_	Path selection sensitivity coefficient	0.5
*q*	Capacity of link	2000pcu/h	*ρ* ^*pc*^	Private Car fuel cost	0.8 yuan/km
*v*_*f*_	Walking speed	5km/h	*ε*	-	0.0001

The proposed NSGA-II algorithm is used. The maximum number of iterations of the algorithm is set to 100, and the initial number of populations is 50. Some Pareto optimal solution sets of the operation strategy model are shown in Table 6.

**Table 6:** Pareto optimal solution set scheme.

Order	Site selection scheme	Pricing scheme	Optimal benefit value
1	{0 1 1 0 1 1 0 0 13 15 0 10 14 0}	{0.85 0.50}	2698
2	{0 0 1 1 1 0 1 0 0 19 14 15 016}	{0.65 0.65}	2826
3	{1 1 0 1 0 1 1 11 13 0 15 0 10 14}	{0.53 0.45}	2878
4	{0 1 1 1 1 1 0 0 18 12 16 13 15 0}	{0.61 0.63}	2890
5	{1 0 1 1 1 0 1 20 0 19 13 12 0 11}	{0.60 0.42}	2961

Table 7 shows site investment intensity and traffic accessibility of each alternative carsharing site in the city.

**Table 7:** Investment intensity and traffic accessibility of alternative sites.

Carsharing sites	2	3	4	5	6	7	9
Site investment intensity (yuan/m^2^)	313	338	390	0.0365	428	361	427
Driving accessibility of site	1.2829	1.2011	1.1325	1.0988	1.0275	0.9566	0.8911

The original evaluation data of each scheme are shown in Table 8.

**Table 8:** Original data table for each scheme.

Scheme	Optimal benefit value (yuan)	Site investment intensity	Driving accessibility of site
1	2760	0.152	4.318
2	2835	0.161	4.150
3	2896	0.180	5.431
4	2900	0.188	5.417
5	2978	0.192	5.433

According to the entropy weight TOPSIS method, the final relative closeness of each scheme is calculated in Table 9.

**Table 9:** Calculation results of relative closeness of each scheme.

Scheme	${\text{D}}_{\text{i}}^{+}$	${\text{D}}_{\text{i}}^{-}$	C^i^
1	0.2512	0.1544	0.3775
2	0.1369	0.3259	0.7016
3	0.1534	0.2533	0.6185
4	0.3651	0.1809	0.3301
5	0.1288	0.3733	0.7405

**Fig 7:**
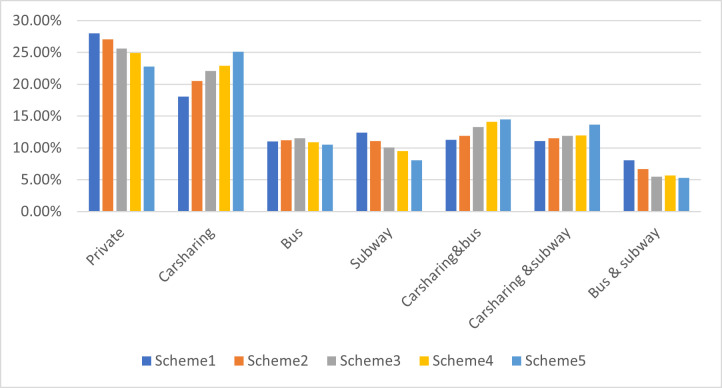
Comparison chart of traffic mode sharing rates in different schemes.

This study considers the economic benefits of operators and the social benefits of the government from the perspective of the system, and supposes that the two benefits account for 50% respectively. As can be seen from Table 6, five different site selection schemes, pricing schemes, and benefit values are obtained under this weighting. The scheme comparison based on entropy weight TOPSIS is used to get the optimal strategy. The calculation results of the relative closeness of each scheme are shown in Table 9, and Scheme 5 has the best value. The share rates of each traffic mode in the five schemes are shown in Table 10 and Fig 7.

**Table 10:** Traffic mode sharing rates in different schemes.

Travel model		Mode Shares				
		Scheme1	Scheme2	Scheme3	Scheme4	Scheme5
Single mode of travel	Private	28.00%	27.10%	25.60%	24.90%	22.80%
	Carsharing	18.10%	20.50%	22.10%	22.90%	25.10%
	Bus	11.00%	11.20%	11.50%	10.90%	10.50%
	Subway	12.40%	11.10%	10.10%	9.50%	8.10%
Combined mode	Carsharing & bus	11.30%	11.90%	13.30%	14.10%	14.50%
	Carsharing & subway	11.10%	11.50%	11.90%	12.00%	13.70%
	Bus & subway	8.10%	6.70%	5.50%	5.70%	5.30%

The following variations of traffic mode sharing in the five schemes can be visualized in Fig 7. The private carsharing rate decreases from Scheme 1 to Scheme 5 due to the adoption of the chosen operating strategy, where the shared cars take up a portion of the traffic volume. In particular, the carsharing rate of Scheme 5 is 25.10%, and the private carsharing rate is reduced to 22.80%. Scheme 5 has the highest sharing rate compared to the other schemes because of the increased deployment and size of carsharing sites, and lower price. Travelers choose carsharing and carsharing + public to travel conveniently and save money, thus reducing the competitiveness of private cars.

Analysis of the combined travel share in Fig 7 shows that the percentage of carsharing + public has also been increased. Scheme 5 has the highest carsharing + public sharing rate among the five schemes. In the designed carsharing strategy, sites are not only located at public transport interchange points but also have sufficient vehicles, so the scheme shortens the waiting time for travelers transferring to public transportation. Shared + public becomes more competitive in the multi-modal transportation system.

In summary, Scheme 5 meets the optimal economic benefits and social benefits, that helps maintain the regular operation of carsharing enterprises and the need for sustainable expansion. Most carsharing sites are located at public transport interchanges, promoting the use of public transportation, and improving the system’s efficiency. It’s consistent with the current policy of giving priority to public transportation.

## Conclusions

This study focuses on how to scientifically design operational strategy that responds to the needs of travelers in multi-modal transportation systems and consider the interests of multiple actors. Firstly, a bi-level programming model for carsharing strategy is developed to maximize economic benefits and social benefits in a multi-mode transportation system. Economic benefits are important to guarantee that the enterprises can balance income and expenditure and make profit, while social benefits are essential to fulfill the needs of the government and users, for sustainable development. Secondly, the site location, number, size, and pricing are jointly planned when designing the carsharing operation strategy. Joint planning overcome the difficulty of achieving the optimal solution for the whole system by considering the site location, number, and pricing separately. Moreover, because the operation strategy is designed in a multi-modal transportation network, we can obtain the impact of the developing shared vehicles on the share rate of travel modes and the change in urban travel structure. It is helpful for traffic managers to understand travel behavior of travelers after the operation of carsharing. Eventually, the case study shows that the site location, number, size, and pricing scheme impact benefit significantly. In the optimal scheme, carsharing operators set reasonable prices, sites are primarily located at public transport interchanges, and there are sufficient vehicles. Here, the sharing rate of private cars is minimal, and the waiting time for transferring to public transport is shortened, so sharing and public transport are more competitive in the transport system. This designed strategy increases the use of shared cars, which helps operators increase revenue, expand market size, promote public transportation use, and improve the system’s efficiency.

Future works will be carried out to optimize the one-way carsharing system by establishing a dynamic traffic allocation model to describe the change of vehicles at a certain period. In addition, the differentiated pricing of OD pairs will be considered.

## Supporting information

S1 Table Data support file. (DOCX)
