# Supplementary material for: Carsharing operation optimization with the comprehensive consideration of economic and social benefits
Source: PLoS One. 2025 Feb 18;20(2):e0315323. doi: 10.1371/journal.pone.0315323 (PMC11835248; doi:10.1371/journal.pone.0315323)
Supplement: S1 Table — (DOCX) [file pone.0315323.s001.docx]

**Table 1.** **Road network information**

| **Road link** | **Link length (km)** | **The car has zero flow time (min)** |
| --- | --- | --- |
| (1,2) | 4 | 5 |
| (2,3) | 6 | 8 |
| (4,5) | 5 | 5 |
| (5,6) | 6 | 8 |
| (7,8) | 6 | 7 |
| (8,9) | 5 | 5 |
| (1,4) | 8 | 11 |
| (2,5) | 6 | 8 |
| (3,6) | 8 | 12 |
| (4,7) | 5 | 6 |
| (5,8) | 6 | 8 |
| (6,9) | 6 | 7 |

**Table 2. Virtual Link information**

| **Link owning set** | **Stretch properties** | **Road link** | | **Link length (m)** |
| --- | --- | --- | --- | --- |
| Set of online links | Private car | $(R,(7,pc))$ | | 0 |
|  | Carsharing | $(R,\left( 7,sc \right))$ | | 200 |
|  | Bus | $(R,\left( 7,pt \right))$ | | 300 |
|  | Subway | $(R,\left( 4,pm \right))$ | | 1200 |
| Set of interchange links | Carsharing transfers with bus | $\{\left( 2,sc \right),(2,pt)\}$ | $\{\left( 2,pt \right),(2,sc)\}$ | 80 |
|  |  | $\{\left( 3,sc \right),(3,pt)\}$ | $\{\left( 3,pt \right),(3,sc)\}$ | 50 |
|  |  | $\{\left( 4,sc \right),(4,pt)\}$ | $\{\left( 4,pt \right),(4,sc)\}$ | 45 |
|  |  | $\{\left( 5,sc \right),(5,pt)\}$ | $\{\left( 5,pt \right),(5,sc)\}$ | 55 |
|  |  | $\{\left( 6,sc \right),(6,pt)\}$ | $\{\left( 6,pt \right),(6,sc)\}$ | 50 |
|  |  | $\{\left( 7,sc \right),(7,pt)\}$ | $\{\left( 7,pt \right),(7,sc)\}$ | 70 |
|  |  | $\{\left( 9,sc \right),(9,pt)\}$ | $\{\left( 9,pt \right),(9,sc)\}$ | 60 |
|  | Carsharing transfers with subway | $\{\left( 3,sc \right),(3,pm)\}$ | $\{\left( 3,pm \right),(3,sc)\}$ | 60 |
|  |  | $\{\left( 4,sc \right),(4,pm)\}$ | $\{\left( 4,pm \right),(4,sc)\}$ | 60 |
|  |  | $\{\left( 5,sc \right),(5,pm)\}$ | $\{\left( 5,pm \right),(5,sc)\}$ | 45 |
|  |  | $\{\left( 6,sc \right),(6,pm)\}$ | $\{\left( 6,pm \right),(6,sc)\}$ | 50 |
|  | Bus transfers with subway | $\{\left( 3,pt \right),(3,pm)\}$ | $\{\left( 3,pm \right),(3,pm)\}$ | 200 |
|  |  | $\{\left( 4,pt \right),(4,pm)\}$ | $\{\left( 4,pm \right),(4,pm)\}$ | 150 |
|  |  | $\{\left( 5,pt \right),(5,pm)\}$ | $\{\left( 5,pm \right),(5,pm)\}$ | 180 |
|  |  | $\{\left( 6,pt \right),(6,pm)\}$ | $\{\left( 6,pm \right),(6,pm)\}$ | 200 |
| Set of off-grid links | Private | $(\left( 3,pc \right),S)$ | | 0 |
|  | Carsharing | $(\left( 3,sc \right),S)$ | | 200 |
|  | Bus | $(\left( 3,pt \right),S)$ | | 500 |
|  | Subway | $(\left( 3,pm \right),S)$ | | 500 |

**Table 3. Parameter value table of the upper model**

| **Parameter** | **Implication** | **Unit** | **Value** |
| --- | --- | --- | --- |
| $\varphi_{1}$ | Maintenance cost per mile traveled | yuan/km | 0.1 |
| $\varphi_{2}$ | The scheduling cost | yuan/veh | 10 |
| $u$ | Site fixed construction costs | yuan | 3$\times{10}^{4}$ |
| $v_{1}$ | Construction cost of a single parking space | yuan | 2$\times{10}^{3}$ |
| $v_{2}$ | Vehicle acquisition cost | yuan | 5$\times{10}^{4}$ |
| $W$ | Maximum acceptable cost for the operator | yuan | 5$\times{10}^{6}$ |
| $\gamma^{c}$ | Coefficient of time value | - | 1.0 |
| $\varphi_{sc}$ | Emissions per kilometer produced by a single carsharing | $kg/(veh\cdot km)$ | 0.213 |
| $\varepsilon_{sc}$ | The amount of ${CO}_{2}$per kilometer produced by a single carsharing | $kg/(veh\cdot km)$ | 0.248 |
| $\delta_{{NO}_{x},CO}$ | Economic loss caused by waste gas emitted per unit of mass | $\mathrm{yuan}/kg$ | 78.54 |
| $\delta_{CO_{2}}$ | Economic loss caused by emission unit mass ${CO}_{2}$ | $yuan/kg$ | 0.23 |
| $\xi_{sc}$ | Fuel consumption of carsharing | $L/(veh\cdot km)$ | 10 |
| $\psi$ | The price per liter of fuel | $yuan/L$ | 8.0 |

**Table 4. Parameter value table of the underlying model**

| **Parameter** | **Implication** | **Value** | **Parameter** | **Implication** | **Value** |
| --- | --- | --- | --- | --- | --- |
| $\alpha$ | - | 0.15 | $\beta$ | - | 4 |
| $\omega_{1}$ | Currency-time conversion coefficient | 2 | $\omega_{2}$ | Comfort-time conversion coefficient | 0.5 |
| $\rho_{w}$ | Comfort loss per unit waiting time | 0.2 | $\rho_{f}$ | Comfort loss per unit walking time | 1 |
| $\lambda_{1}$ | Mode selection sensitivity coefficient | 0.1 | $\lambda_{2}$ | Path selection sensitivity coefficient | 0.5 |
| $q$ | Capacity of link | 2000pcu/h | $\rho^{pc}$ | Private Car fuel cost | 0.8 yuan/km |
| $v_{f}$ | Walking speed | 5km/h | $\varepsilon$ | - | 0.0001 |

**Table 5. Shared car transportation mode information**

| **Parameter** | **Implication** | **Value** | **Parameter** | **Implication** | **Value** |
| --- | --- | --- | --- | --- | --- |
| $\lambda$ | Average arrival rate of users | 200per/h | $\mu$ | Average usage rate of users | 15min |
| $s$ | Acceptable walking distance | 500m | $b$ | Average number of passengers carried | 3per/pcu |
| $\rho_{l}^{sc}$ | Mileage rate | 0.9 yuan/km | $\rho_{t}^{sc}$ | Time rate | 0.4 yuan/min |
| $v$ | free flow speed | 40km/h |  |  |  |

**Table 6.** Public transportation mode information

| **Mode of transportation** | **Departure interval**  **(min)** | **Passenger capacity**  **(per)** | **Driving speed**  **(km/h)** | **Fare**  **(yuan)** | **Comfort loss per unit travel time** |
| --- | --- | --- | --- | --- | --- |
| Bus | 15min | 30 per | 25km /h | 2 yuan | 0.5 |
| Metro | 10min | 500 per | 60km/h | 3 yuan | 0.3 |
